# Oral corrective feedback in English as a foreign language classrooms: A teaching and learning perspective

**DOI:** 10.1016/j.heliyon.2021.e07550

**Published:** 2021-07-12

**Authors:** Xuan Van Ha, Loc Tan Nguyen, Bui Phu Hung

**Affiliations:** a Department of Foreign Languages, Ha Tinh University, Ha Tinh, Viet Nam; b School of Foreign Languages, University of Economics Ho Chi Minh City, Ho Chi Minh City, Viet Nam

**Keywords:** Oral corrective feedback, Feedback timing, Feedback types, Teacher beliefs, Learner beliefs, EFL

## Abstract

Oral corrective feedback, a key topic in second language pedagogy and research in applied linguistics and second language acquisition, has widely been investigated for the past two decades. However, the relationship between teachers' and students' beliefs about oral corrective feedback has been relatively underexplored. The current study extends this line of research by examining the extent to which Vietnamese English as a foreign language teachers' and students' beliefs concerning the importance, types, and timing of feedback are aligned. The data consisted of questionnaires with 250 students, interviews with 15 of those who completed the questionnaires, and interviews with 24 teachers at four public secondary schools in Vietnam. The findings showed some matches and mismatches between the teachers' and students' beliefs. Both the teachers and students highly valued the efficacy of feedback and were positive about explicit feedback types such as explicit corrections and metalinguistic feedback. Regarding feedback timing, the students preferred immediate feedback while the teachers expressed their concerns about the students' emotional state and the possibility of disruption of immediate feedback on the flow of students' speech. The findings are interpreted in relation to sociocultural factors, contextual factors, and teachers' and students’ experiences. Implications for language teachers, teacher educators, and professional development program designers are discussed.

## Highlights

• Both teachers and students are positive about feedback efficacy and necessity.
• Both teachers and students are positive about explicit feedback types.
• Students value immediate feedback, while teachers prefer to delay their feedback.
• Teacher and learner beliefs are influenced by sociocultural and contextual factors.
• Teacher and learner beliefs are influenced by their experiences.

## 1 Introduction

Beliefs, as defined by Borg (2011), are “propositions individuals consider to be true […] which are often tacit, have a strong evaluative and affective component, provide a basis for action, and are resistant to change” (pp. 370–371). Teachers' and students' beliefs are essential factors that mediate both the process and outcomes of teaching and learning (Borg, 2015; Ellis, 2008). In second or foreign language (L2) teaching and learning, previous research has revealed that teachers' and students' beliefs are not always congruent (Ellis, 2008; Ha and Nguyen, 2021; Jean and Simard, 2011; Nguyen and Newton, 2019). For example, Brown (2009) found that the teacher participants appreciated a communicative orientation in teaching while the students preferred a grammar-oriented learning approach. In a recent study, Ha and Nguyen (2021) revealed that students preferred to receive feedback for all error types and wished to be trained to provide peer feedback, while teachers were more selective in their choices of feedback targets and were sometimes sceptical about their students' ability to do peer correction. These mismatches between the teachers' and students' beliefs may result in adverse effects on the behaviours and outcomes of teaching and learning. According to Ellis (2008), teachers should “make their own beliefs about language learning explicit, to find out about their students' beliefs, to help their students become aware of and to evaluate their own beliefs and to address any mismatch in their and their students' belief systems” (p. 24). The past few decades have seen considerable research attention regarding teacher and learner beliefs within the context of English as a second/foreign language (ESL/EFL) education (Borg, 2015; Calafato, 2020; Phipps and Borg, 2009). However, the relationship between teachers' and students’ beliefs concerning corrective feedback has been under-researched.

Corrective feedback, a fundamental part of teaching and learning in various L2 classrooms (Ha and Murray, 2021; Lyster et al., 2013), has triggered the interest of both L2 teachers and researchers in applied linguistics and second language acquisition (SLA) (Ellis, 2017; Ha and Nguyen, 2021; Lyster et al., 2013). It can be provided either in an oral mode (e.g., teachers' oral responses to learners' spoken errors) or a written mode (e.g., teachers' written comments on students' written assignments). Both oral corrective feedback (OCF) and written corrective feedback have been shown to be effective for learners’ L2 development (Ellis, 2009; Li and Vuono, 2019). However, these two modes of feedback “have unique features and have been examined separately in the primary research” (Li and Vuono, 2019, p. 94). Within the scope of this article, only OCF was investigated; therefore, any mention of feedback in this current study refers to OCF.

Beliefs about OCF merit more research attention because this line of inquiry can provide more insights into the (in)congruence between teachers' and students' beliefs. This understanding can, in turn, help teachers enhance the efficacy of their OCF provision. Contemporary literature shows that most of the previous studies have been conducted with adult learners, especially those in the ESL contexts of western countries. But much less research has investigated teachers' and students' beliefs about OCF in EFL contexts, especially at the secondary level. Notably, to the best of our knowledge, there is a paucity of research exploring teachers' and students’ OCF beliefs in secondary EFL contexts in Asian countries, including Vietnam. The current study is, therefore, timely in order to address this research gap by investigating the beliefs of Vietnamese secondary EFL teachers and students regarding three aspects of OCF, namely, the OCF importance, types, and timing.

## 2 Literature review

### 2.1 Oral corrective feedback

OCF, defined as teachers' or peers' responses to learners' erroneous utterances, has received extensive research attention for the past two decades. Most of the previous research investigating the effectiveness of OCF has shown OCF to be beneficial and necessary for L2 learners' language development (Li, 2010; Lyster et al., 2013; Mackey and Goo, 2007; Nassaji, 2016, 2017). Research on the feedback frequency and patterns in L2 classrooms suggests that feedback frequently occurs in many classroom events (Brown, 2016; Ha, 2017; Lyster and Ranta, 1997; Sheen, 2004; Wang and Li, 2020). In Lyster and Ranta's (1997) seminal work, they identified six main feedback types in French immersion classrooms, namely explicit corrections, recasts, elicitation, repetition, clarification requests, and metalinguistic feedback. Lyster and Mori (2006) later grouped these feedback types into three broader categories: recasts, explicit corrections, and prompts. Following the feedback taxonomy of Lyster and his colleagues, many studies have looked into the patterns and effectiveness of various feedback types, revealing that recasts were most frequently used by teachers, but prompts elicited more immediate learner uptake (Brown, 2016; Wang and Li, 2020). Studies employing a pre-test and post-test design revealed that all feedback types were effective (Li, 2010; Lyster and Saito, 2010; Nassaji, 2017), and the efficacy of feedback depends on a number of mediating factors such as individual learner differences, the manner of delivery, and the conditions in which feedback is provided (Nassaji and Kartchava, 2020).

### 2.2 Teachers' and students’ beliefs concerning the importance, types, and timing of oral corrective feedback

Most of the research investigating OCF beliefs has been conducted as part of larger projects that focus on teachers' and students' beliefs concerning language learning and teaching. These studies usually included several questionnaire items eliciting teachers' and students' views on the efficacy or/and need of OCF as part of a broader survey. This body of research shows that students were generally much more positive about the efficacy and necessity of OCF than teachers (Brown, 2009; Jean and Simard, 2011; Loewen et al., 2009; Schulz, 1996, 2001). The main reason for this discrepancy was ascribed to teachers' concern about students' emotional well-being and the possibility of disruption of OCF (Kartchava et al., 2020; Li, 2017; Roothooft and Breeze, 2016). L2 learners' desire for OCF has been found to be dependent on the learning context and their previous learning experiences (Ha et al., 2021; Loewen et al., 2009). Research has also suggested that students' beliefs are one of the factors that mediate learners' uptake following feedback and learners’ noticing of the corrective function of feedback (Akiyama, 2017; Kartchava, 2019; Kartchava & Ammar, 2014).

In terms of teachers' and/or students' beliefs about OCF types, several studies have been carried out in a certain range of contexts and showed some mixed findings. Research by Lee (2013), for instance, showed that advanced ESL learners in the US ranked explicit corrections as their favourite type of feedback and metalinguistic feedback as their least preferred type. This finding is inconsistent with previous research which showed that metalinguistic feedback was preferred by most secondary and tertiary ESL students in Singapore (Oladejo, 1993). In a study involving 395 learners (both adults and teenagers) and 46 teachers in the Spanish EFL context, Roothooft and Breeze (2016) showed that the learners were more willing to receive explicit kinds of OCF such as explicit corrections and metalinguistic feedback while the teachers were reserved to use these feedback types but preferred a more implicit type such as elicitation. Also, the teachers were concerned about the possible negative reactions from students, while the students did not seem to believe so. Zhang and Rahimi's (2014) research, which involved 160 Iranian adult EFL learners (80 high anxiety and 80 low anxiety learners), showed that the learners strongly favoured metalinguistic feedback and explicit corrections regardless of their anxiety levels. By contrast, in Zhu and Wang's (2019) study within the Chinese tertiary EFL context, the learner participants reported that they favoured prompts (e.g., repetition and metalinguistic feedback) rather than explicit corrections. Overall, these studies suggest that students' preferences for feedback types are influenced by the teaching and learning contexts, and EFL students tend to be more inclined to receive metalinguistic feedback than ESL students in the US. Students seem to be positive about explicit feedback, but teachers are less positive due in part to their concern about students' affective responses to feedback. However, it remains unknown about the feedback preferences of students and teachers in secondary EFL contexts in Asia, including Vietnamese secondary schools.

Another strand of research focusing on teachers' and/or students' beliefs concerning OCF rests on the ideal timing for teachers to correct students' erroneous utterances. OCF can be immediate or delayed. Immediate OCF is provided more or less as soon as an error occurs, while delayed OCF does not take place until a pedagogical activity which serves as a context for correction has been completed (Li et al., 2016). Davis (2003), in a study with 97 EFL students and 18 teachers in Macau, found that 86% of the students but only one-third of the teachers (6/18) reported that errors should be corrected more or less as soon as they were made to help students avoid forming bad habits. Research by Brown (2009), which involved 49 teachers and 1,409 university ESL students in the US, revealed that the teachers were more supportive than the students of the idea that effective teachers should not use immediate feedback. In contrast, Iranian university EFL students in Zhang and Rahimi's (2014) study preferred immediate to delayed feedback, and Chinese tertiary EFL students in Zhu and Wang's (2019) research also expressed a negative attitude towards delayed feedback. The teacher participants in Ha and Murray's (2020, 2021) qualitative studies were also found to be sceptical about the workability of immediate feedback. Generally, this line of research suggests that students are more positive about immediate OCF than teachers, but further studies are needed to gain a more conclusive understanding of teachers' and students' perspectives about feedback timing. This research evidence is important as it can help to inform more definite pedagogical implications in this regard.

In short, the studies reviewed above have demonstrated some tentative conclusions regarding the beliefs of teachers and students about different aspects of OCF. However, research investigating teachers' and students' beliefs concerning feedback types and feedback timing is limited. While teachers' and students' feedback beliefs have been shown to be influenced by the teaching and learning context, this research focus in secondary EFL settings that include a large L2 learner population is underexplored. There is, therefore, a need for more research to gain more nuanced insights into teachers' and students’ beliefs concerning various aspects of OCF in a more varied range of contexts. And the current study is a timely one. It seeks to address the following three research questions:

1. What are Vietnamese EFL teachers' and students' beliefs concerning the role of oral corrective feedback?
2. What are Vietnamese EFL teachers' and students' preferences for oral corrective feedback types?
3. What are Vietnamese EFL teachers' and students' preferences for oral corrective feedback timing?

## 3 Methods

This study employed a mixed-methods research design to investigate the relationship between teachers' and students' beliefs concerning OCF in Vietnamese secondary EFL classrooms. Data included three sources, namely students' questionnaires, students' follow-up interviews, and teachers’ interviews. The data collection and analysis procedures strictly followed the guidelines established in the ethics approval.

### 3.1 Contexts

The settings of this study included four upper secondary schools in a central Vietnamese province. Each school employed 10–13 English teachers and had 36–45 classes, with 30–45 students in each class. In Vietnam, English is a mandatory foreign language subject that is taught over three lessons (45 min each) per week. English is included in the final high-stakes exams for graduation. According to the national curriculum, secondary students are expected to obtain a preliminary level of English proficiency upon graduation (equivalent to level B1, Common European Framework of Reference for Languages) upon graduation (MOET, 2010). However, in reality, English teaching and learning approaches are highly exam-oriented (Ha and Murray, 2021). Exams are usually in written formats which test students’ knowledge of English vocabulary and grammar. Oral assessment is rarely, if not never, applied.

### 3.2 Participants

Convenience sampling was employed to recruit participants based on willingness and availability. A total of 250 students, including 98 males and 152 females, completed a questionnaire. Of these, 15 students (seven males and eight females) were invited to participate in a follow-up interview, and pseudonyms (Student 1 – Student 15) were used for the sake of confidentiality. The students were between 15 and 18 years of age. Most of them had their first English lessons from Grade 6, and some from Grade 3. The classroom was the primary site for students' exposure to the target language, although some were able to take extra English lessons in their out-of-class time, at school, private language centres, or their teachers’ houses. The English proficiency of the students ranged from elementary to intermediate level.

A total of 24 teachers (all Vietnamese) coming from the four schools volunteered to participate in the study, including one male and 23 females. Within the four selected schools for the current study, only three out of the 47 English teachers were males. This ratio of male to female teachers reflects the reality of the English language teaching workforce distribution in Vietnam. The teachers had between 10 and 21 years of experience (mean = 15.8 years) in teaching English at secondary schools. They had all obtained a bachelors’ degree in teaching EFL before starting their career. Regarding English proficiency, the teachers all passed the English proficiency test for secondary English teachers required by the Ministry of Education and Training. For ethical issues, the teachers were given pseudonyms (Teacher 1 – Teacher 24) in this report.

### 3.3 Instruments

The instruments for data collection comprised a questionnaire for students, a list of guiding questions for students' semi-structured interviews, and a list of guiding questions for the teachers’ interviews. These instruments were developed by the researchers for a broader project of which the current investigation is a part.

The questionnaire was constructed based on an extensive synthesis of research on learner beliefs concerning OCF (e.g., Kartchava & Ammar, 2014; Loewen et al., 2009; Schulz, 1996, 2001) following rigorous procedures of piloting and validation as discussed in some questionnaire construction guidelines (e.g., Dörnyei and Taguchi, 2009). The questionnaire was initially developed in English. It was subjected to various rounds of revision and polishing via meetings and discussions among the research team members. As the students’ English proficiency may not be sufficient to ensure the most insightful responses, we decided to translate the questionnaire into Vietnamese before administering it to the students. The translation was conducted by the first author and was then cross-checked for accuracy by two bilingual colleagues (Vietnamese and English). Next, content validation was carried out separately with three teachers and five students before the questionnaire was piloted. The validation was performed through group discussions where the teachers and students were invited to discuss openly any concerns, hesitations, or feedback with the researcher concerning both the content and wording of the questionnaire items. Following the comments of the students and teachers, amendments were made with some items. The questionnaire was then piloted with 100 students from two schools which were not the main study setting. The students participating in the pilot study and those in the main study were similar regarding age, learning conditions, and proficiency levels. The pilot study results enabled the researchers to exclude some flawed items to improve scale reliability. Satisfactory reliability was obtained (α = .83).

The questionnaire's final version comprised two parts, namely demographic questions and the questions about students' beliefs (main part). The main part included 47 Likert-scale items which focused on the students' beliefs concerning various aspects of OCF in L2 classrooms. The Cronbach's alpha value for the main study was .85, illustrating good internal consistency for the instruments (Dörnyei and Taguchi, 2009). Within the scope of the current study, items eliciting the students' beliefs about (1) the role of OCF (Q1 – Q7), (2) types of OCF (Q35 – Q44), and (3) timing of OCF (Q11 – Q17) were used.

Students' interviews were conducted to help the researchers elaborate on and further interpret the quantitative results from the questionnaire data. Guiding questions for semi-structured interviews were developed based on two sources of reference, namely, the preliminary results of the students’ questionnaires and the synthesis of the OCF literature.

Teachers' beliefs about OCF were elicited through semi-structured interviews. The guiding questions were developed with reference to a comprehensive synthesis of OCF research. Following Ha and Murray's (2020) suggestion, OCF types were elicited via several steps to achieve a nuanced understanding of teachers' beliefs. Firstly, the teachers were provided with three OCF scenarios to discuss the necessity of feedback and the feedback strategies they would rely on (if any) in such scenarios. Secondly, they were given a sample of 11 OCF examples for each scenario to discuss the benefits and drawbacks of each example in each scenario. Once the teachers were familiar with the OCF types, many of which they might have used before, they were requested to comment on their general views on and preferences for feedback types. Regarding feedback timing, the teachers were first asked to comment on the benefits and drawbacks of the four times of feedback: (1) as soon as an error is made, (2) after an utterance has been completed, (3) after the activity, and (4) by the end of the lesson. They were then asked to elaborate on their beliefs and preferences concerning feedback timing.

### 3.4 Data collection and analysis

Firstly, the teachers were interviewed individually in the staff room at their schools by the first author. Each interview session lasted from 63 to 78 min and was audio recorded with a digital recorder. Secondly, 250 students were provided with paper-based questionnaires to complete at their convenience. At the end of the questionnaire was an item asking the students for willingness to participate in a follow-up interview. One week later, 247 questionnaires were returned. Of these, 11 questionnaires were removed from the data set because they were incomplete. The final data set for analysis included 236 complete questionnaires. To ensure feasibility, we invited the first 15 volunteers to participate in interviews. The students' interviews were conducted individually by the first author three weeks after the students had completed the questionnaires. On average, each interview session lasted for about 22 min and was audio recorded. Considering the pros and cons of the choice of the interview language (Cortazzi et al., 2011), we decided to conduct both the teachers' and students’ interviews in Vietnamese to avoid any possible language difficulties and to maximise understanding between the interviewer and interviewees. Quotes reported in the current article were translated into English.

Regarding the analysis of the questionnaire, descriptive statistics were used to investigate the students’ beliefs about the importance, types, and timing of OCF with the support of SPSS software. The interview data were analysed thematically (Braun and Clarke, 2006) with the help of NVivo software. First, all the interviews with the students and teachers were transcribed verbatim by the researchers. Second, the transcripts were read several times for a complete understanding of the data. Third, phrases and sentences which have similar meanings were classified into categories. The codes were then revised and refined to develop broader themes. The following themes were reported and discussed in this current paper: (1) the OCF efficacy and necessity, (2) OCF types, and (3) OCF timing.

## 4 Results

### 4.1 Feedback efficacy and necessity

Overall, the students were very positive about the role of OCF, with the overall mean score being over 4.0 out of 5.0 for every item except for Item 6. As shown in Table 1, of the seven items eliciting students' beliefs about the role of OCF, the two items receiving the highest mean scores were those that focused on the importance of OCF in facilitating students' learning (Q1, mean = 4.48) and on students' overall desire for OCF (Q3, mean = 4.42). The item asking students’ desire for OCF while they were doing group work activity received the lowest mean score (Q6, mean = 3.89).

**Table 1:** Students’ beliefs about the role of OCF.

	N	Min	Max	Mean	SD
Q1. Teachers' corrective feedback (teachers' response to students' spoken errors) is important for students' English learning.	236	1	5	4.48	.642
Q2. Teachers' corrective feedback helps students to consolidate their English speaking.	236	1	5	4.35	.714
Q3. If I make an error, I want my teacher to correct it.	236	1	5	4.42	.787
Q4. If I make an error when I am answering my teacher's question, I want my teacher to correct it.	236	1	5	4.31	.744
Q5. If I make an error when I am presenting something in English to the whole class, I want my teacher to correct it.	236	1	5	4.12	.806
Q6. If I make an error when I am talking in a group-work activity, I want my teacher to correct it.	236	1	5	3.89	.925
Q7. If I make an error related to the focus of the lesson, my teacher should correct it.	236	1	5	4.19	.830

Analysis of the interview data revealed that all of the students believed OCF to be essential for their learning. Two-thirds of the interviewed students (10/15) expressed a wish to be corrected as much as possible. They reasoned that they were used to it because their teachers corrected them frequently. They commented that teachers' OCF could help them improve their language accuracy, which was necessary for exams. Some students even said that teachers' OCF was a requirement of the teachers' job. For example, Student 2 said, “correcting errors is the teacher's main job; it is just like teaching or explaining the rules of grammar or meanings of vocabulary”.

Analyses of the teachers' interview data showed that all 24 teachers were generally positive about OCF. They considered that errors are part of students’ language learning and that OCF is integral to their teaching activities. For example, Teacher 1 said:

Oral corrective feedback is very necessary for students' learning. It's normal for students to make errors, and our job is to help students correct their errors to improve their accuracy in speaking, writing, and improving their exam results. We can ignore some errors, but basically, we have to correct students' errors.

The teachers gave some comments on the necessity of OCF for particular situations. They estimated that OCF should be provided for about 30%–80% of students' errors, depending on students’ proficiency level, teaching activity, and the lesson focus. They explained that given the practical contextual constraints, including teaching time and class size, OCF should be selective. For example, Teacher 3 commented:

Giving oral corrective feedback is indispensable. However, how often I do corrections depends on the stage of the lesson. For example, in the post-task stage, I usually encourage students to talk as much as possible. I want them to talk freely without having to worry about making errors. By contrast, in a while-task activity, students need to practise using the language so that I correct them very often.

Some teachers also mentioned that teachers needed to pay attention to students' well-being in making OCF decisions. They expressed a concern that correcting a particular student too frequently may adversely influence their emotional state. For example, Teacher 18 stated, “It's not a good idea to correct too much, especially when focusing on one student. He/she may feel fed up with teachers' feedback or may lose confidence. This will adversely influence their participation in further activities”.

### 4.2 Feedback types

Table 2 shows the students' beliefs about the main types of OCF as elicited via ten items (Q35–Q44). As seen in Table 2, metalinguistic feedback received the highest mean score (Q37, mean = 4.12), followed by integrated recasts (Q42, mean = 4.03), interrogative recasts (Q43, mean = 4.00), and explicit corrections (Q44, mean = 3.95). It can be inferred from the descriptive statistics of these items that the students preferred either to be provided with the correct answers to their erroneous utterances or to receive teachers' explanations about the language rules. Of all other three subtypes of prompts, repetition received the highest mean (Q36, mean = 3.94). Notably, clarification requests received the lowest level of approval (Q38, mean = 3.46). Teachers' use of body language or gestures as a way of identifying students’ errors also received a low level of approval (Q39, mean = 3.47).

**Table 2:** Students’ preferences for OCF types.

	N	Min	Max	Mean	SD
Q35. If I make an error, I want my teacher to say my utterance again and pause before the error so that I can correct it by myself (e.g. I…).	236	1	5	3.75	1.011
Q36. If I make an error, I want my teacher to repeat my erroneous utterance with a change in intonation so that I can recognise the error and correct it by myself, or my friends can correct it (e.g., I go?).	236	1	5	3.94	.950
Q37. If I make an error, I want my teacher to give me comments or language rules so that I can correct it by myself or my friends can correct it (e.g., You need the past tense).	236	1	5	4.12	.839
Q38. If I make an error, I want my teacher to ask me to say the utterance again such as ‘What?/What did you say?/Or can you say it again?’	236	1	5	3.46	.938
Q39. If I make an error, I want my teacher to use his/her body language or gestures to signal that there is an error so that I can correct it by myself, or my friends can correct it.	236	1	5	3.47	.915
Q40. If I make an error, I want my teacher to give me the correct form by repeating the whole utterance and reformulating the erroneous part (e.g., I went to the train station yesterday).	236	1	5	3.70	.939
Q41. If I make an error, I want my teacher to give me the correct form by reformulating and repeating only the erroneous part of the utterance (e.g., I went).	236	1	5	3.70	.925
Q42. If I make an error, I want my teacher to give me the correct form by reformulating the erroneous part and ask me another short question (e.g., You went to the train station yesterday. Did you meet someone there?	236	1	5	4.03	.850
Q43. If I make an error, I want my teacher to reformulate the erroneous utterance and put it in the form of a confirmation check or a question (e.g., Where did you say you went yesterday?).	236	1	5	4.00	.904
Q44. If I make an error, I want my teacher to tell me explicitly that there is an error and give me the correct form (e.g., No, not ‘go’, you should say ‘went’).	236	1	5	3.95	.957

These beliefs were confirmed in the students’ interviews. Most of the students who took part in the interviews (12/15) reported preferring metalinguistic comments and elicitations the most because they helped them understand the errors and have a chance to self-correct. The students also said that metalinguistic feedback was a very frequent OCF type that their teachers used. Three students cited that clarification requests were not preferred because they caused confusion and worries. As Student 10 commented:

I don't feel confident when my teacher says, “sorry, can you say it again?” or “what?”. These kinds of sentences make me worried because I don't know what's going on. I don't even know that my teacher would like me to self-correct my sentences.

Analyses of the teachers’ interview data revealed that the teachers had different views from their students regarding OCF types. Before the teachers were given OCF type samples to discuss, they stated that they provided feedback unconsciously and intuitively without much consideration of related factors. They believed that explaining to students the language rules underlying their errors was the most important for students to learn from their errors. For instance, Teacher 10 said:

I usually give my students some clues or comments on their errors, such as “this should be an adjective, not a noun”. I need to explain to them so that they can understand why they make the errors and remember the language rules for future uses.

After the teachers had been given examples of various OCF types for the three scenarios to discuss, they provided some more in-depth comments regarding their preferences for feedback types. The teachers expressed some tensions between the ideal feedback type and the practicality with their teaching contexts. They said that they sometimes had to trade off between feedback types to suit their classroom reality. Overall, 16 out of the 24 teachers preferred to use prompts. Seven teachers reported that explicit corrections were their preferred feedback type because they informed students of the errors and provided students with the correct forms. Only one teacher chose recasts as her favourite feedback type since recasts were quick and easy for her students.

The teachers further explained that they wanted to make their feedback explicit to raise students’ awareness of its corrective nature. Also, they believed that students could learn better if they had a chance to self-correct their erroneous utterances. According to the teachers, self-correction could involve students in deeper processing of the language rules, making them “think about their errors and the correct forms” (Teacher 10). All the teachers were convinced that recasts were quick and easy to administer and easy for their students; however, they were concerned that recasts might not be salient enough for their students to notice their corrective nature.

### 4.3 Feedback timing

Students' beliefs about the ideal time for receiving OCF were examined via seven items. As shown in Table 3, the students generally preferred immediate feedback to delayed feedback. Some relationships between students' preferences for feedback timing and error types were identified. Specifically, questions asking the necessity of immediate feedback for errors influencing communication (Q15) and errors related to the lesson focus (Q16) received the highest levels of approval. Q13 and Q14 focused on students’ preferences for delayed feedback and received relatively low levels of approval. Interestingly, Q17 asking whether feedback for less important errors should be delayed received a very low level of approval (2.38/5.0). This suggests that the students did not prefer delayed feedback even when the errors were less important.

**Table 3:** Students’ preferences for OCF timing.

	N	Min	Max	Mean	S.D
Q11. I want my teacher to correct me as soon as I make an error.	236	1	5	3.54	1.161
Q12. My teacher should wait and correct my error after I have finished speaking.	236	1	5	3.74	1.030
Q13. My teacher should note my error down or remember it then correct it in front of the class at the end of the lesson.	236	1	5	3.16	1.227
Q14. My teacher should wait until the end of the activity that I am involved in to correct my error.	236	1	5	3.25	1.133
Q15. If I make an error which can interfere with my teacher's or peers' understanding, my teacher should correct it immediately.	236	1	5	3.88	1.049
Q16. If I make an error related to the grammar focus or the new vocabulary of the lesson, my teacher should correct it immediately.	236	1	5	3.90	1.026
Q17. If I make an error which is NOT important, my teacher should leave it and correct it later.	236	1	5	2.38	1.067

In the interviews, most of the students reported that they would like to receive immediate feedback and elaborated on their preferences for feedback timing. Typically, all of the students considered that immediate feedback was good because it could help them realise their errors immediately. On the other hand, they did not highly value delayed feedback since they may have forgotten what they said or what errors they made. Accordingly, they did not have a chance to repeat their teachers’ reformulations. For example, Student 12 commented:

I like to be corrected as soon as I make an error because it will help me to know what is wrong with my speaking. I may forget everything after I speak, so it's not helpful if my teacher does not correct it straightaway.

On the question of how immediate teachers' feedback should be, most of the students said that they would like to receive teachers’ feedback immediately after they completed a sentence or an utterance, rather than delaying feedback either to the end of the activity or until the end of the lesson. Four students gave more in-depth comments, claiming that feedback timing should depend on error types; for example, complicated errors should be delayed until after the activity had been completed.

Regarding the teachers' views about feedback timing, they all held a belief that feedback should be provided after students finished their speaking or at the end of the teaching activity. They elaborated by saying that giving students feedback while they were speaking could negatively influence their emotional state and discourage their future participation in classroom learning. Therefore, from the teachers’ perspective, correcting after students finished their speaking was a teaching principle which could not be challenged, as evidence in the following comments:

Teacher 15: I never correct my students while they are speaking. I wait until they finish speaking, even until all students finish speaking, to give feedback at the same time. Correcting while students are doing their speaking activity will make them embarrassed and lose confidence.

Teacher 6: I never correct immediately after students' errors. Doing so can disrupt students' talk and make them forget what they are speaking.

The teachers also considered the ideal OCF timing in relation to error types. For example, Teacher 19 said, “In cases where a student has difficulty with what to say, I can provide the correct word straightaway. In other cases, such as students mispronouncing words or misusing grammar, I will wait until the end to correct.” The teachers claimed to support students to speak as much as possible.

Some teachers commented that immediate OCF could be suitable for students' accuracy work (activity focusing on developing students' accuracy) because the interactions usually comprised short questions and answers and immediate feedback may not influence the flow of students’ speech. For example, Teacher 3 said, “if a student speaks only one sentence and makes an error, I can correct it immediately after the sentence”. By contrast, in fluency work (activity focusing on fluency development), the teachers believed that OCF should be delayed until the activity had been completed.

## 5 Discussion

One of the most notable findings of the current study was that both the teachers and students had a positive attitude towards OCF. The finding that students were positive about OCF is aligned with previous research (e.g., Ha et al., 2021; Kim and Mostafa, 2021; Li, 2017; Zhang and Rahimi, 2014), but that the teachers were very positive about OCF is different from some previous studies conducted in western ESL contexts (e.g., Brown, 2009; Li, 2017; Schulz, 1996, 2001). Recent research has shown that students generally expressed a positive attitude towards OCF while teachers were hesitant to provide OCF due to their concern about the possibility of causing students' embarrassment or anxiety (Li, 2017; Roothooft and Breeze, 2016). This difference may be explained in relation to teaching and learning contexts. Most of the previous studies were conducted in ESL contexts, the teaching focus of which prioritised developing students' communicative competence rather than explicit language knowledge or language accuracy for written exam purposes. By contrast, both the teachers and students in our study seemed to prioritise the development of language accuracy for their subsequent exams. This suggests that the teaching and learning in the contexts of the current study were influenced by the washback effect of high-stakes exams. This finding aligns with previous studies involving Vietnamese EFL teachers. For example, Ha and Murray (2020, 2021) found that Vietnamese EFL teachers were positive about OCF because they had been teaching in exam-oriented environments and provided OCF frequently as an integral part of their teaching job. In the current study, similar comments were found in the interviews with both the teacher and student participants. Another possible reason for the teachers’ positive attitudes towards OCF lies with their teaching experience. Research has shown that the more experienced the instructors are, the more positive their attitude is towards OCF (Kim and Mostafa, 2021; Rahimi and Zhang, 2015). In our study, the fact that all the teacher participants had more than 10 years of teaching experience might account for why they were so positive about OCF.

Regarding feedback types, there was some congruence between the teachers' and students' beliefs in that they both liked metalinguistic feedback and highly appreciated the effectiveness of this feedback type. This finding is contradictory to that of Lee's (2013) study with advanced ESL learners in the US but lends support to previous studies in Chinese EFL (Zhu and Wang, 2019) and Iranian EFL (Zhang and Rahimi, 2014) contexts. A possible explanation for this finding is the effects of the test-driven teaching and learning approaches in the Vietnamese EFL context (Ha, 2017; Ha and Nguyen, 2021). Both the students and teachers highly valued the explanations of language rules for improving students' explicit knowledge of grammar and vocabulary as they would help enhance the results of subsequent high-stake exams which primarily assessed students' knowledge of grammar and vocabulary (Ha & Murray, 2020, 2021). This may also account for the teachers' and students' preferences for explicit corrections, which enabled students to recognise their errors and receive the correct forms. As with other subtypes of prompts, both the students and teachers stated that they liked elicitation and repetition. Both groups of participants explained in the interviews that they appreciated the value of self-correction because it could facilitate deeper processing and learning.

Notably, the teachers were very positive about OCF and were inclined to employ explicit types of feedback such as explicit corrections and metalinguistic feedback. This finding is different from that of some previous studies (Basturkmen et al., 2004; Kamiya, 2016; Roothooft and Breeze, 2016). The teachers' positive attitude towards OCF, in general, and explicit feedback types, in particular, may be influenced by the teachers’ experience (Rahimi and Zhang, 2015) and the exam-oriented teaching contexts (Ha & Murray, 2020, 2021). This may also be explained by the traditional Vietnamese educational role relationship where teachers are considered experts or knowledge givers and students as knowledge receivers (Ha and Murray, 2020; Ha and Nguyen, 2021). Thus, teachers are expected to give students the correct answers.

Another notable finding of the current study is the difference between the teachers' and students' preferences for feedback timing. The students preferred to receive feedback more or less as soon as they made an error in most situations, while the teachers wanted to delay their feedback until after a speaking activity or by the end of the lesson. This finding is consistent with that of previous research (Brown, 2009; Davis, 2003). As explained by most of the students in the interviews, they were not concerned about the possible negative effects of immediate feedback. Still, they wished to receive feedback as soon as their utterances had been completed so that they would not forget what they had been speaking and had a chance to repeat the correct forms. The teachers, however, perceived that correcting students' errors on the spot was appropriate for accuracy work only, but not for fluency work. According to the teachers, this belief had been shaped in their professional development activities. It may also be influenced by the popular teachers’ guides (e.g., Harmer, 2007). Ellis (2017) points out a research – pedagogy gap between SLA researchers and L2 methodologists regarding perspectives on feedback timing. Ellis also argues that these methodologists may need to revise their teacher guides by referring to updated SLA research findings for a research-based pedagogy.

Despite the contributions discussed above, several limitations of the current study need to be acknowledged. Firstly, beliefs about OCF have been found to be dynamic (Kim and Mostafa, 2021; Leontjev, 2016); therefore, a one-shot questionnaire and interviews may not capture the complexity and dynamics of teachers' and students’ OCF beliefs. Thus, future studies may need to employ a longitudinal approach such as asking the participants to keep a diary of their views and experiences over several semesters or to conduct a series of interviews over an extended period to depict a complete picture of OCF beliefs. Secondly, the practical constraints did not allow this study to employ a random sampling method and recruit a larger number of participants, limiting the generalizability of the findings. As such, it is important to consider these caveats when interpreting the current study findings.

## 6 Conclusion

This study investigated Vietnamese teachers' and students' beliefs concerning OCF in secondary EFL settings, revealing some matches and mismatches between the teachers' and students' beliefs. Both groups of participants shared similar preferences for feedback types and believed that OCF was beneficial and necessary for learning and teaching. However, the teachers' and students’ beliefs about the ideal feedback timing were not congruent in that the students favoured receiving feedback immediately when errors occurred, but the teachers would like to delay their feedback until after the activity or by the end of the lesson.

As alluded to above, the teachers' extensive teaching experience, the exam-oriented teaching and learning contexts, and the traditional Vietnamese educational role relationship between students and teachers might account for the findings that the teachers were very positive about OCF and were disposed to use explicit feedback types. The teachers' beliefs about feedback timing may be influenced by some teachers' guides and their previous experiences in teacher education and professional development. The conflicting view about OCF timing has been reported as a current research–pedagogy gap (Nassaji, 2012; Sato and Loewen, 2019). Specifically, SLA researchers suggest that immediate feedback works for both accuracy and fluency work, while teachers tend to be reserved about immediate feedback (Ellis, 2017). Therefore, the findings of this study can be a relevant source of reference for L2 teachers and teacher educators to reflect on. As suggested by Ellis (2008), teachers may need to consider students’ beliefs and find ways to openly discuss them with their students to avoid mismatches in beliefs. On the basis of the study findings, it might be necessary that designers of language education and teacher professional development programs consider incorporating a belief component regarding OCF, and beyond, so that teachers can be aware of and reflect on their beliefs while teaching or engaging in professional development activities.

## Declarations

### Author contribution statement

Xuan Van Ha: Conceived and designed the experiments; Performed the experiments; Analyzed and interpreted the data; Contributed reagents, materials, analysis tools or data; Wrote the paper.

Loc Tan Nguyen, Bui Phu Hung: Analyzed and interpreted the data; Wrote the paper.

### Funding statement

This work was funded by University of Economics Ho Chi Minh City.

### Data availability statement

Data will be made available on request.

### Declaration of interests statement

The authors declare no conflict of interest.

### Additional information

No additional information is available for this paper.
